# High-resolution prediction and risk mapping of *Haemaphysalis longicornis* habitat suitability in the Jiaodong Peninsula, China

**DOI:** 10.1016/j.isci.2026.117723

**Published:** 2026-09-22

**Authors:** Xue-Kun Bian, Shi-Pan Chen, Jia-Yi Xu, Han Xue, Ao-Long Xu, Shi-Jun Song, Ying-Zhi Chong, Fei Wang, Na Tian, Hao-Ran Ding, Jing-Han Feng, Yan Lu, Lan-Hua Li

**Affiliations:** 1 School of Public Health, Shandong Second Medical University, Weifang, Shandong 261053, People's Republic of China; 2 School of Medicine, Qingdao Binhai University, Qingdao, Shandong 266555, People's Republic of China; 3 National Institute of Parasitic Diseases, Chinese Center for Disease Control and Prevention, Chinese Center for Tropical Diseases Research, National Key Laboratory of Intelligent Tracking and Forecasting for Infectious Diseases, Key Laboratory on Parasite and Vector Biology, Ministry of Health, WHO Collaborating Centre for Tropical Diseases, National Center for International Research on Tropical Diseases, Ministry of Science and Technology, Shanghai 200025, People's Republic of China

**Keywords:** tick, species distribution prediction, machine learning, ensemble model, Jiaodong Peninsula, China

## Abstract

Ticks are vectors of numerous human pathogens, and accurate predictions of their spatial distribution are essential for the effective prevention and control of tick-borne diseases. High-resolution predictions remain scarce in China. To address this gap, a tick survey was conducted across the Jiaodong Peninsula at 2,647 points during the summer of 2024, integrating high-resolution environmental variables. Seven machine-learning algorithms were used, and ensemble learning was applied, resulting in a habitat-suitability map for *Haemaphysalis longicornis* at a 10-meter resolution. The ensemble model showed robust predictive accuracy (AUC = 0.8355). Summer normalized difference vegetation index was the primary environmental driver, followed by land use and land cover and winter normalized difference vegetation index. High risk villages accounted for 70.0%, 40.2%, and 16.9% of rural, urban rural fringe, and urban areas, respectively. Ensemble machine-learning provides a robust approach, revealing how ecological factors and human activities shape distribution patterns and risk heterogeneity.

## Highlights

• First 10-m resolution ensemble map of *H. longicornis* suitability in Jiaodong Peninsula
• Large-scale field survey and multi-source data drive robust model
• Over 61% of communities at high risk, rural areas most vulnerable, urging targeted control

## Graphical abstract

**Figure:**
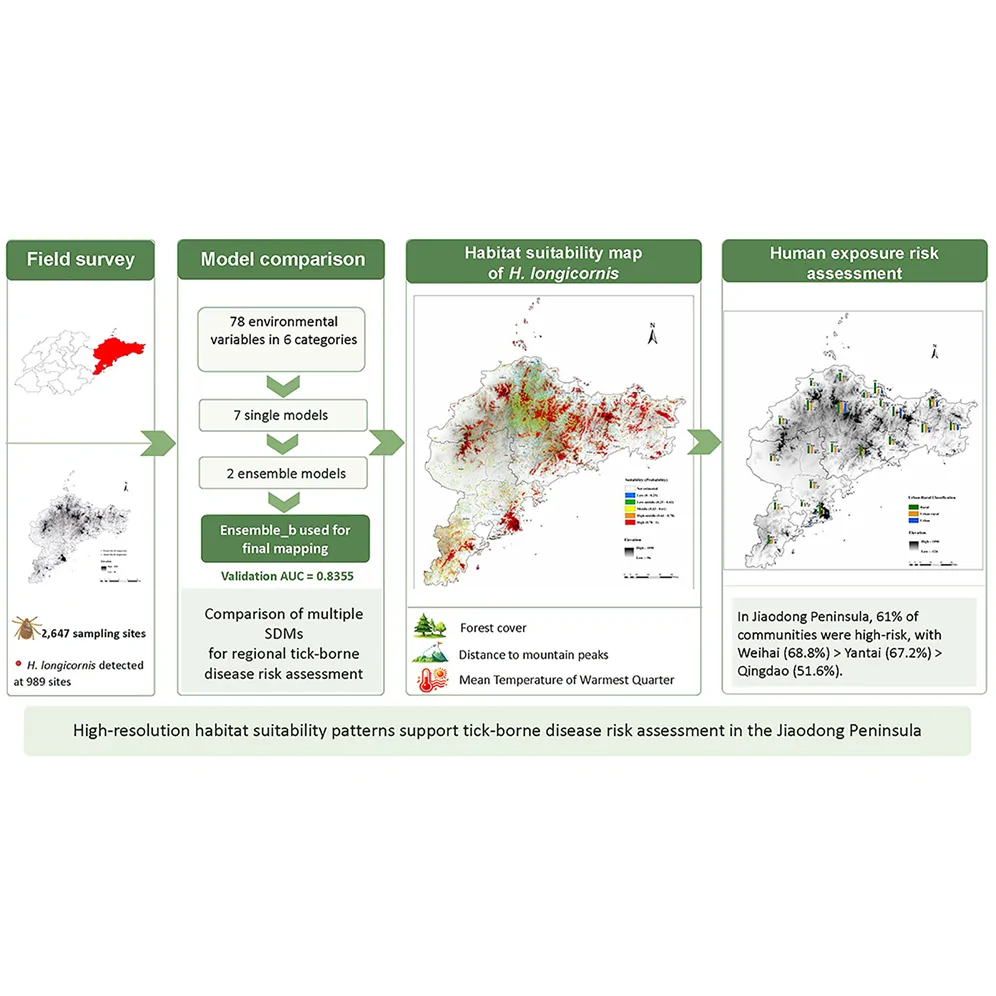

## Teaser

Public health; Environmental health; Entomology

## Introduction

Ticks are obligate hematophagous ectoparasites that infest a broad spectrum of vertebrates, including reptiles, birds, mammals, and amphibians. Globally distributed and closely linked to human and animal health, ticks are capable of transmitting a variety of bacterial, viral, and parasitic pathogens, ranking as the second most important vector of human diseases after mosquitoes.^1^^,^^2^ Diseases such as Lyme disease, tick-borne encephalitis, and severe fever with thrombocytopenia syndrome (SFTS) continue to impose a substantial public health burden worldwide.^3^^,^^4^

China is among the countries significantly affected by tick-borne diseases and faces unique ecological drivers that may influence transmission risk. Since the mid-1990s, nationwide afforestation and ecological restoration programs have substantially increased the extent and quality of forest and grassland coverage.^5^ While beneficial for biodiversity and ecosystem services, these habitat modifications have expanded suitable environments for tick hosts and likely contributed to the enlargement of favorable tick habitats, potentially exacerbating the risk of tick-borne pathogen transmission in certain regions. In recent years, the number of tick-borne pathogens reported in China has risen, along with an increase in reported cases of diseases such as SFTS, indicating a growing public health challenge.^6^^,^^7^

Obtaining high-resolution spatial data on tick distribution is fundamental to establishing effective surveillance, early-warning systems, and targeted control strategies. Globally, numerous studies have utilized “presence-only” records extracted from historical literature or surveillance databases to model tick and pathogen distributions.^8^ However, such data are often spatially coarse, lacking precise geographic coordinates and frequently approximated to administrative-unit centroids. This spatial uncertainty hampers the integration of high-resolution environmental variables—such as land use and vegetation structure—that are critical for characterizing habitat heterogeneity. Consequently, most existing models rely on macro-climatic predictors and generate coarse-scale suitability maps, which offer limited insight into local-scale distribution patterns. Relying on historical data for research, areas with missing or sparse data are not necessarily those with a low probability of tick presence; they may instead be areas with low survey effort. Such bias may have a significant impact on prediction results.

In contrast, a limited number of studies have employed “presence-absence” data collected through systematic field surveys.^9^ While these improve spatial accuracy, they are often constrained by small sample sizes, which may compromise model performance and generalizability. In China, tick distribution modeling based on systematic, field-collected data remains scarce. Most existing studies lack the sample density necessary to support reliable fine-scale predictions, limiting their practical utility in public health planning and intervention.^1^^,^^10^

*Haemaphysalis longicornis* is the dominant tick species on the Jiaodong Peninsula and is capable of harboring more than ten tick-borne viruses, including SFTS virus (SFTSV) and various subtypes of tick-borne encephalitis virus (TBEV), among others.^6^ Meanwhile, *Haemaphysalis flava* and *Haemaphysalis doenitzi* are found in low numbers on the peninsula, and studies have demonstrated that *H. flava* can carry Jingmen tick virus.^11^ The Jiaodong Peninsula, located in eastern Shandong (119°30′–122°42′E, 35°35′–38°23′N), encompasses the cities of Qingdao, Yantai, and Weihai. The region has a warm-temperate continental monsoon climate, covers approximately 31,022.94 km^2^, and is characterized by mountainous and hilly terrain accounting for about two-thirds of its area. Forest land exceeds 5,000 km^2^, and orchard coverage surpasses 3,475 km^2^. These environmental conditions support diverse wildlife communities and provide suitable ecological settings for tick survival and reproduction.

In this study, we aim to produce a high-resolution map predicting *H. longicornis* ticks habitat suitability across the Jiaodong Peninsula. Through field surveys of more than 2,000 transects, integration of multi-source high-resolution environmental variables, and application of machine-learning ensemble modeling, we seek to provide a spatially explicit tool to support tick-borne disease risk assessment, surveillance optimization, and precision prevention.

## Results

### Overview of the field survey

From July to August 2024, a total of 2,647 sampling points were investigated across the Jiaodong Peninsula. *Haemaphysalis longicornis* ticks were detected at 989 points (37.36%); additionally, 207 points were infested with *Haemaphysalis flava*, and 162 points with *Haemaphysalis doenitzi*. Among different habitat types, the highest detection rate occurred in evergreen forests (50.00%, 160/320), followed by broad-leaved forests (49.89%, 695/1,393), grasslands (13.27%, 82/618), and orchards (16.46%, 52/316). In total, 8,019 ticks were collected, comprising 6,084 *H*. *longicornis* (2,138 adults and 3,946 nymphs), 1,338 *H. flava* (18 adults and 1,320 nymphs), and 597 *H. doenitzi* (513 adults and 84 nymphs).

Based on these occurrence records, this study presents the predicted distribution of *H. longicornis*. Because only a limited number of occurrence records were available for *H. flava* and *H. doenitzi* (207 and 162 points, respectively, compared to 989 points for *H. longicornis*), we did not conduct separate predictions for these species.

Spatially, *H*. *longicornis* presence and absence points were intermingled without pronounced regional segregation (Figure 1), indicating that sampling effectively covered diverse environmental gradients and avoided systematic bias toward high-risk zones. Topographic analysis revealed that although *H*. *longicornis*-present sites occurred throughout the peninsula, they were more frequent in the hilly and mountainous areas with marked relief in the northern and central-eastern regions, exhibiting a patchy, aggregated pattern. In contrast, *H*. *longicornis*-absent sites were more concentrated in lower-altitude, flatter terrain.

**Figure 1:**
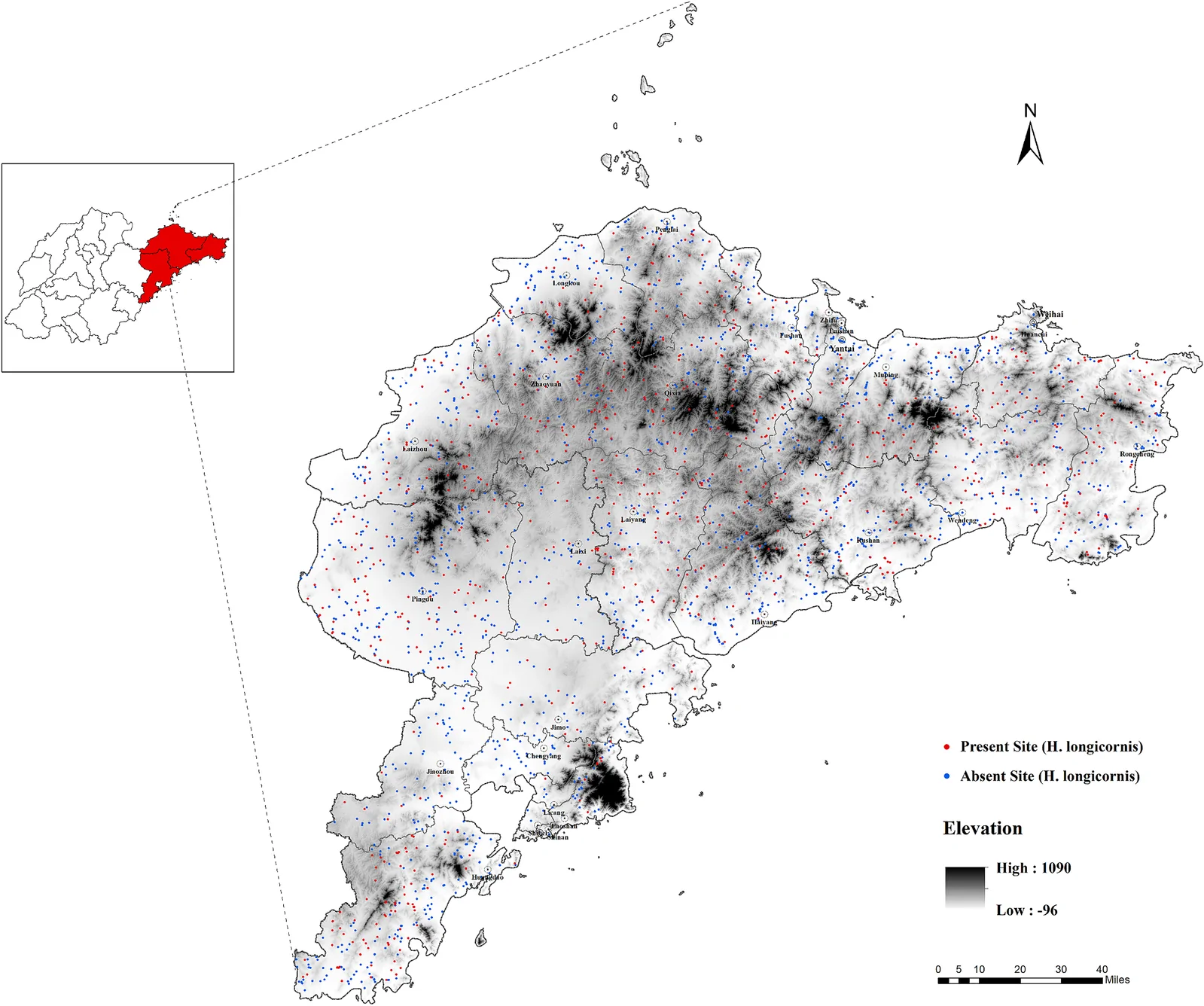
Topography and spatial distribution of tick sampling points across the Jiaodong Peninsula

### Model performance and selection of the optimal model

After variable screening via LASSO regression combined with Spearman correlation analysis, 25 environmental predictors were retained for modeling (Table S1). Seven individual machine-learning models and two ensemble models were constructed; their performance metrics are summarized in Table 1.

**Table 1:** Performance comparison of individual and ensemble models on training, test, and validation sets

Model	AUC	Threshold	Sensitivity	Specificity	TSS	F1_score	Brier_score
**Training set**							

RF	0.9638	0.3907	0.9480	0.8233	0.7712	0.8462	0.1072
GBM	0.9038	0.3636	0.8579	0.7757	0.6336	0.7697	0.1274
GLM	0.8413	0.3806	0.7961	0.7264	0.5225	0.7079	0.1574
MARS	0.8631	0.3512	0.8228	0.7443	0.5670	0.7326	0.1457
XGBoost	0.9207	0.4525	0.8312	0.8394	0.6706	0.7928	0.1183
NNet	0.8524	0.3982	0.8031	0.7392	0.5423	0.7187	0.1521
MaxEnt	0.8705	0.5393	0.7792	0.7935	0.5727	0.7347	0.1511
Ensemble_a^a^	0.9153	0.3982	0.8622	0.7859	0.6481	0.7779	0.1246
Ensemble_b^b^	0.8872	0.4654	0.7848	0.8165	0.6013	0.7515	0.1360

**Test set**							

RF	0.8433	0.3907	0.8503	0.7093	0.5596	0.7395	0.1633
GBM	0.8245	0.3636	0.7968	0.7059	0.5027	0.7078	0.1683
GLM	0.8314	0.3806	0.8075	0.7059	0.5134	0.7139	0.1631
MARS	0.8251	0.3512	0.8128	0.7197	0.5326	0.7238	0.1667
XGBoost	0.8421	0.4525	0.7380	0.7751	0.5131	0.7077	0.1587
NNet	0.8198	0.3982	0.7487	0.7024	0.4511	0.6780	0.1689
MaxEnt	0.8304	0.5393	0.7487	0.7578	0.5064	0.7053	0.1672
Ensemble_a^a^	0.8446	0.3982	0.8021	0.7405	0.5426	0.7282	0.1587
Ensemble_b^b^	0.8403	0.4654	0.7433	0.7889	0.5322	0.7183	0.1593

**Validation set**							

RF	0.8166	0.3907	0.7733	0.6815	0.4549	0.6339	0.1617
GBM	0.8255	0.3636	0.7600	0.7070	0.4670	0.6404	0.1556
GLM	0.8363	0.3806	0.7733	0.7452	0.5186	0.6705	0.1529
MARS	0.7936	0.3512	0.7733	0.7134	0.4867	0.6517	0.1749
XGBoost	0.8228	0.4525	0.7200	0.7516	0.4716	0.6429	0.1579
NNet	0.8385	0.3982	0.7600	0.7261	0.4861	0.6514	0.1523
MaxEnt	0.8382	0.5393	0.7067	0.7834	0.4901	0.6543	0.1599
Ensemble_a^a^	0.8291	0.3982	0.7600	0.7134	0.4734	0.6441	0.1550
Ensemble_b^b^	0.8355	0.4654	0.7067	0.7898	0.4965	0.6584	0.1533

a Ensemble_a integrates RF, XGBoost, and GLM.

b Ensemble_b integrates GBM, GLM, MARS, XGBoost, NNet, and MaxEnt.

All models exhibited good discriminative ability on the training set (AUC = 0.8413–0.9638). Performance generally declined on the test and validation sets, suggesting some degree of overfitting. On the test set, the ensemble models (Ensemble_a, Ensemble_b) outperformed the individual models. In the validation set, Ensemble_b achieved the best overall performance (AUC = 0.8355, Brier score = 0.1533).

Overfitting was evident for random forest (RF) (ΔAUC = 0.1205), where the difference between training and test AUC exceeded 0.1. Ensemble_a, which included RF, also showed reduced robustness. In contrast, Ensemble_b displayed no substantial overfitting (ΔAUC = 0.0469) and maintained high discrimination accuracy with low prediction error on the validation set. Therefore, Ensemble_b was selected as the optimal model for subsequent analysis and mapping.

### Contributions of environmental drivers

Detailed descriptions and definitions of all environmental variables are provided in Table 2. Variable importance analysis based on the ensemble model indicated that summer normalized difference vegetation index (NDVI; ndvi_summer) was the most influential single predictor, accounting for 16.22% of the explanatory contribution (Figure 2). Partial dependence plots further illustrated that *H*. *longicornis* occurrence probability showed a positive correlation with this predictor, increasing monotonically with rising values (Figure 3). The third most important variable was winter NDVI (ndvi_winter, 7.02%) (Figure 2). *Haemaphysalis longicornis* occurrence probability also showed a positive correlation with ndvi_winter, increasing monotonically with rising values (Figure 3).

**Table 2:** Candidate environmental predictors and data sources

Category	Variable name	Explanation	Source
Climate	BIO1	annual mean temperature	WorldClim
–	BIO2	mean diurnal range (mean of monthly [max temp-min temp])	WorldClim
–	BIO3	isothermality (BIO2/BIO7) (×100)	WorldClim
–	BIO4	temperature seasonality (standard deviation ×100)	WorldClim
–	BIO5	max temperature of warmest month	WorldClim
–	BIO6	min temperature of coldest month	WorldClim
–	BIO7	temperature annual range (BIO5-BIO6)	WorldClim
–	BIO8	mean temperature of wettest quarter	WorldClim
–	BIO9	mean temperature of driest quarter	WorldClim
–	BIO10	mean temperature of warmest quarter	WorldClim
–	BIO11	mean temperature of coldest quarter	WorldClim
–	BIO12	annual precipitation	WorldClim
–	BIO13	precipitation of wettest month	WorldClim
–	BIO14	precipitation of driest month	WorldClim
–	BIO15	precipitation seasonality (coefficient of variation)	WorldClim
–	BIO16	precipitation of wettest quarter	WorldClim
–	BIO17	precipitation of driest quarter	WorldClim
–	BIO18	precipitation of warmest quarter	WorldClim
–	BIO19	precipitation of coldest quarter	WorldClim
Topography	elevation	elevation	Shuttle Radar Topography Mission (SRTM) digital elevation model (DEM)
–	slop	slope	derived from SRTM DEM
–	aspect	aspect	derived from SRTM DEM
–	TWI	topographic wetness index	derived from SRTM DEM
–	tr1000	topographic relief within a radius of 1,000 m	derived from SRTM DEM
–	coastline	the Euclidean distance to the nearest coastline	China National Geomatics Information Platform (CNGIS)
–	mount	the Euclidean distance to the nearest mountain peak	Resource and Environment Science and Data Center (RESDC)
Land use & human activity	LULC	land use and land cover type	supervised classification based on Sentinel-2
–	forest100	proportion of forest land within a 100-meter radius buffer zone^b^	derived from LULC map
–	forest1000	proportion of forest land within a 1000-meter radius buffer zone^b^	derived from LULC map
–	npp	nighttime light index, proxy for human activity	Visible Infrared Imaging Radiometer Suite - Suomi National Polar-orbiting Partnership (VIIRS-NPP)
Remote sensing ecological index^a^	ndvi_spring/summer/autumn/winter/mean/sd	normalized difference vegetation index	Sentinel-2
–	lst_spring/summer/autumn/winter/mean/sd	land surface temperature	Landsat 8
–	smi_spring/summer/autumn/winter/mean/sd	soil moisture index	Sentinel-1
Radar backscattering characteristics^a^	vv_spring/summer/autumn/winter/mean/sd	VV polarization, Sentinel-1 C-band satellite vertical launch-vertically received back scatter coefficient	Sentinel-1
–	vh_spring/summer/autumn/winter/mean/sd	VH polarization, Sentinel-1 C-band satellite vertical launch-horizontal receive back scatter coefficient	Sentinel-1
–	vvvh_spring/summer/autumn/winter/mean/sd	polarization ratio of VV to VH	Sentinel-1
Image texture characteristics^a^	tc_spring/summer/autumn/winter/mean/sd	texture complexity, the local heterogeneity of inverted pixel values based on the grayscale co-pixel matrix derived from Sentinel-2 imagery	Sentinel-2
–	ts_spring/summer/autumn/winter/mean/sd	texture strength, the local homogeneity of inverted pixel values based on the grayscale co-pixel matrix derived from Sentinel-2 imagery	Sentinel-2

a The remote-sensing ecological indices, radar backscatter, and image texture features were calculated for each season (spring, summer, autumn, and winter); from these seasonal values, two statistics—mean and standard deviation—were derived.

b Forest proportion combines broad-leaved and evergreen forest.

**Figure 2:**
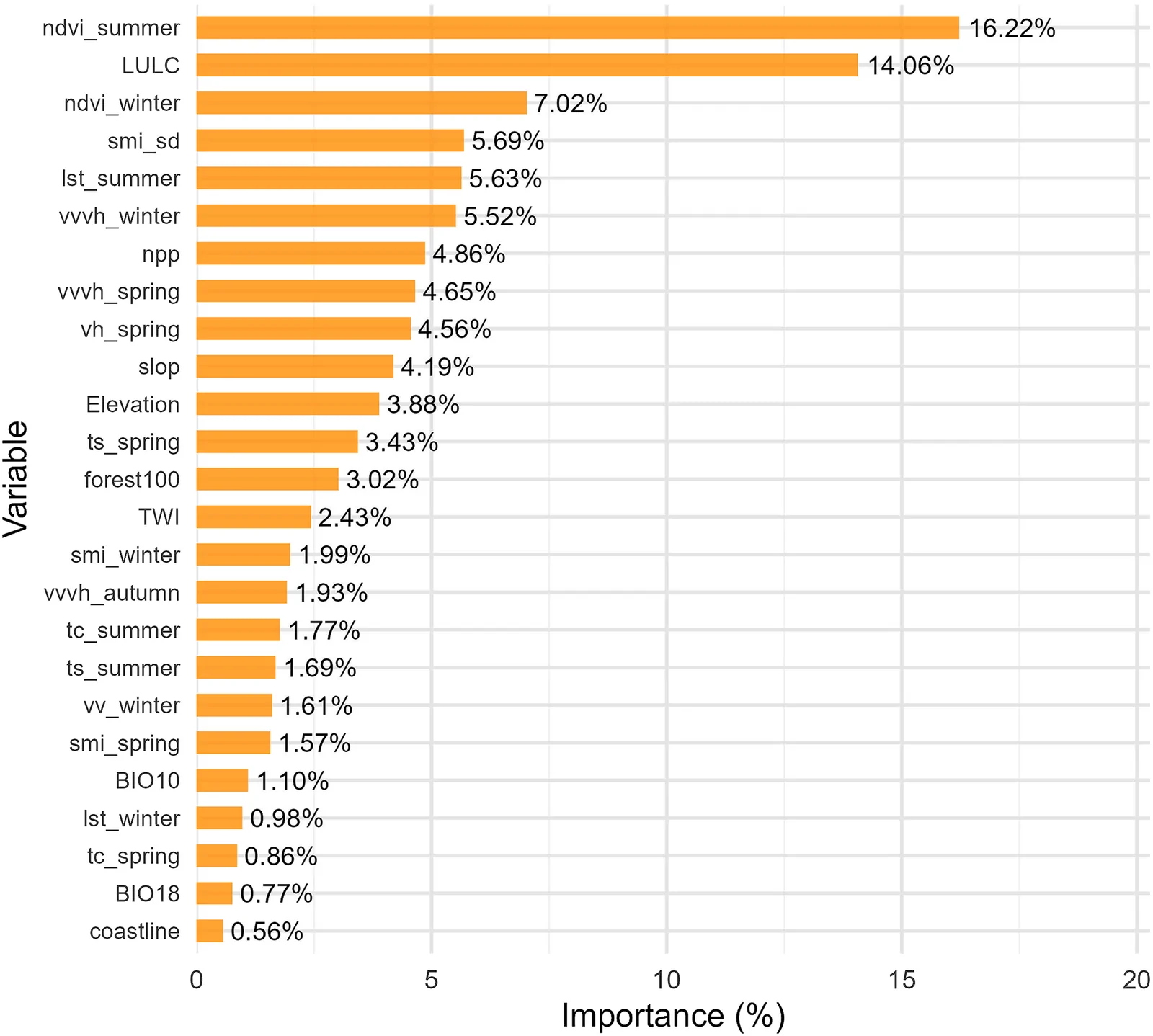
Variable importance ranking of environmental predictors based on the final ensemble model Note: ndvi_summer, ndvi_winter, normalized difference vegetation index; LULC, land use and land cover type; smi_sd, smi_spring, smi_winter, soil moisture index; lst_summer, lst_winter, land surface temperature; vvvh_spring, vvvh_autumn, vvvh_winter, polarization ratio of VV to VH; npp, nighttime light index, proxy for human activity; vh_spring, VH polarization, Sentinel-1 C-band satellite vertical launch-horizontal receive back scatter coefficient; slop, slope; elevation, elevation; ts_spring, ts_summer, texture strength, the local homogeneity of inverted pixel values based on the grayscale co-pixel matrix derived from Sentinel-2 imagery; forest100, proportion of forest land within a 100-meter radius buffer zone; TWI, topographic wetness index; tc_spring, tc_summer, texture complexity, the local heterogeneity of inverted pixel values based on the grayscale co-pixel matrix derived from Sentinel-2 imagery; vv_winter, VV polarization, Sentinel-1 C-band satellite vertical launch-vertically received back scatter coefficient; BIO10, mean temperature of warmest quarter; BIO18, precipitation of warmest quarter; coastline, Euclidean distance to the nearest coastline.

**Figure 3:**
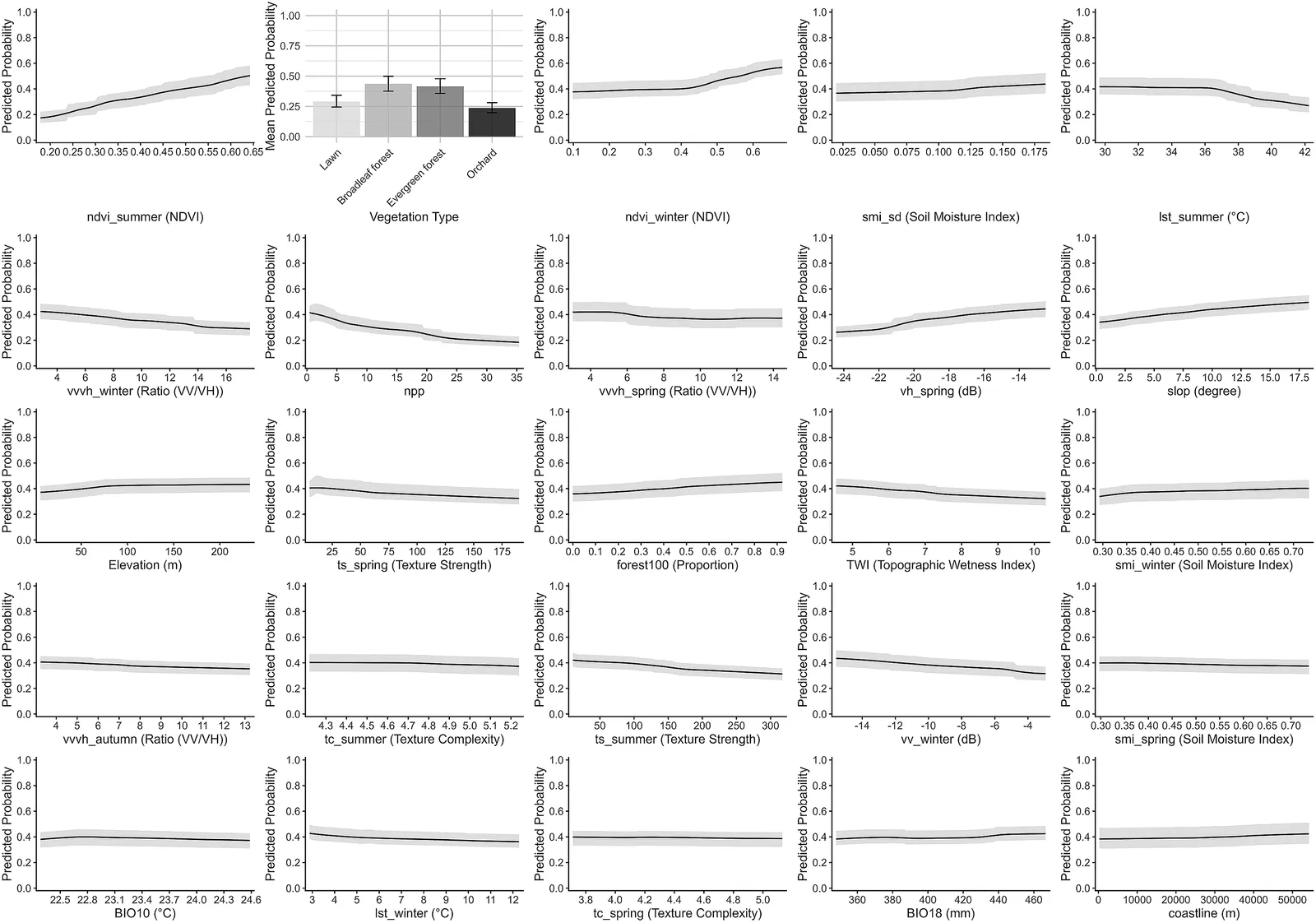
Partial dependence plots illustrating the marginal effects of key environmental variables on *H. longicornis* occurrence probability Note: The plots show the predicted probability (*y* axis) as a function of each variable (*x* axis), with other covariates held at their observed values in the training data. Solid black lines represent mean predicted probabilities averaged across 50 subsamples, each comprising 3% of the training data. Gray shaded areas represent 95% confidence intervals, defined as the 2.5th and 97.5th percentiles of the subsampling distribution. For the categorical variable LULC, bar heights represent mean predicted probabilities for each vegetation type, with error bars showing the 95% confidence intervals. Variable abbreviations and units (where applicable) are as follows: ndvi_summer, ndvi_winter, normalized difference vegetation index (unitless); LULC, land use and land cover type (categorical); smi_sd, smi_spring, smi_winter, soil moisture index (unitless); lst_summer, lst_winter, land surface temperature (°C); vvvh_spring, vvvh_autumn, vvvh_winter, polarization ratio of VV to VH (unitless); npp, nighttime light index, proxy for human activity (unitless); vh_spring, VH polarization backscatter coefficient (dB); slop, slope (degree); elevation, elevation (m); ts_spring, ts_summer, texture strength, the local homogeneity of inverted pixel values based on the gray-scale co-pixel matrix from Sentinel-2 (unitless); forest100, proportion of forest land within a 100-m radius buffer (unitless); TWI, topographic wetness index (unitless); tc_spring, tc_summer, texture complexity, the local heterogeneity of inverted pixel values based on the gray-scale co-pixel matrix from Sentinel-2 (unitless); vv_winter, VV polarization backscatter coefficient (dB); BIO10, mean temperature of warmest quarter (°C); BIO18, precipitation of warmest quarter (mm); coastline, Euclidean distance to the nearest coastline (m).

The next most important variable was land use and land cover (LULC) type, accounting for 14.06% of the explanatory contribution (Figure 2). Partial dependence plots further illustrated that *H*. *longicornis* occurrence probability was markedly higher in broad-leaved and evergreen forests than in grasslands or orchards (Figure 3).

Several other factors provided moderate but stable contributions: standard deviation of soil moisture index (smi_sd, 5.69%), summer land surface temperature (lst_summer, 5.63%), winter VV/VH polarization ratio (vvvh_winter, 5.52%), nighttime light index (npp, 4.86%), spring VV/VH polarization ratio (vvvh_spring, 4.65%), spring VH polarization backscatter (vh_spring, 4.56%), spring texture strength (ts_spring, 3.43%), and topographic variables including slope (4.19%) and elevation (3.88%) (Figure 2). *Haemaphysalis longicornis* occurrence probability was negatively correlated with summer surface temperature, winter VV/VH polarization ratio, nighttime light index and spring VV/VH polarization ratio, and positively correlated with standard deviation of SMI, spring VH backscatter, slope, and elevation (Figure 3). Remaining variables each contributed less than 3% and played auxiliary roles in the model.

### Predicted spatial distribution of *H*. *longicornis* habitat suitability

The probability map of *H*. *longicornis* distribution generated by the final ensemble model was classified into five suitability levels (low, medium-low, medium, medium-high, and high) using the natural breaks method (Figure 4).

**Figure 4:**
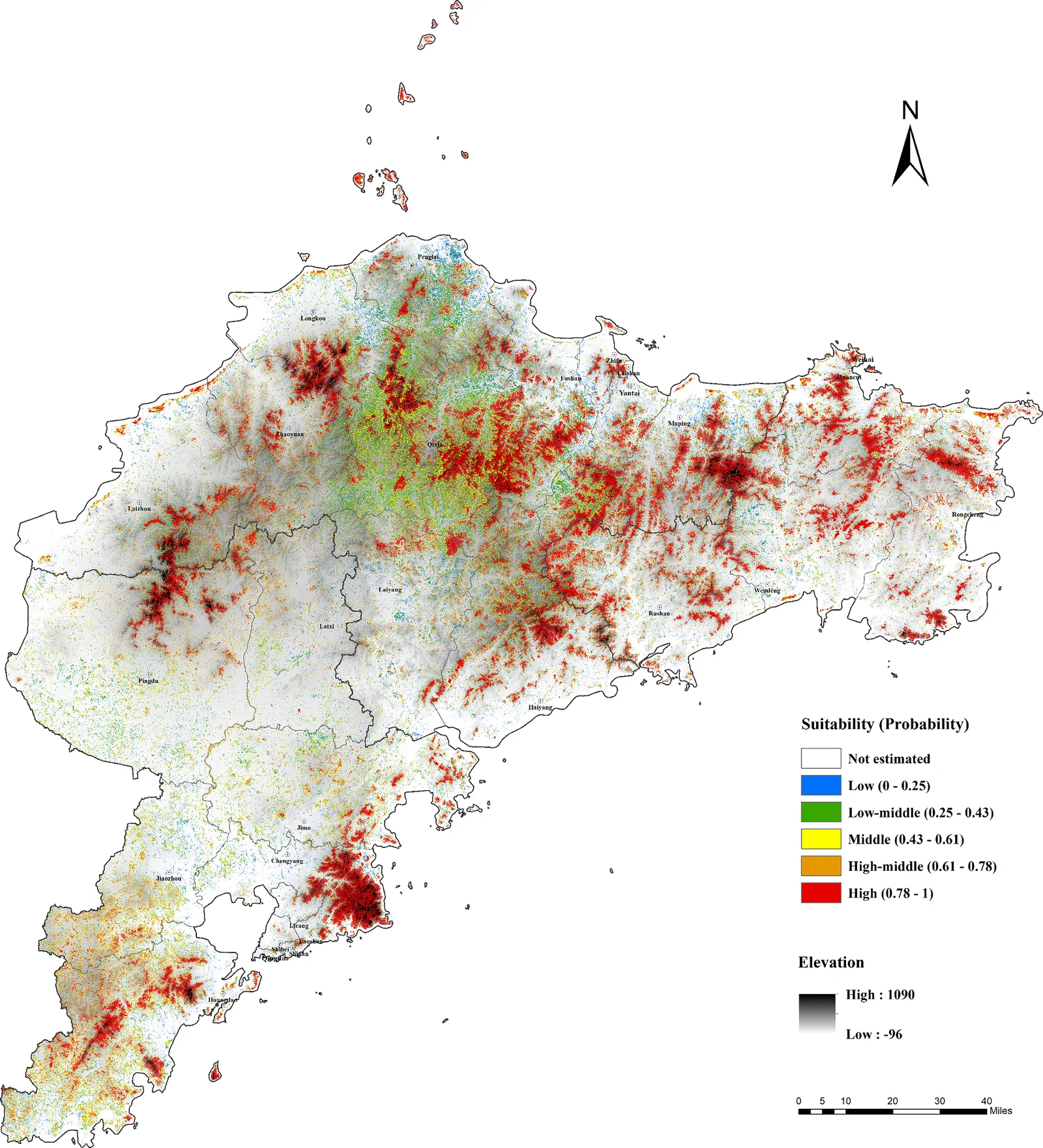
Predicted habitat suitability map for *H. longicornis* in the Jiaodong Peninsula: Classified into five levels based on the final ensemble model

Overall, suitable *H*. *longicornis* habitats in the Jiaodong Peninsula exhibited a spatial structure described as “aggregated along mountain corridors and attenuated toward surrounding lowlands.” High-suitability areas (shown in red) occurred mainly in patches or strips that closely aligned with elevated terrain. In mountainous or hilly regions with pronounced relief, high-suitability patches were densely distributed and well-connected. In low-altitude plains, habitats were predominantly of medium-low or low suitability, with only sporadic medium-high probability points.

Regionally, predicted hotspots were predominantly clustered within the central hilly and mountainous zones spanning Yantai and Weihai. In Yantai, a contiguous swath of medium-high to high suitability extended from the Zhaoyuan-Qixia area eastward into Muping. In Weihai, high-suitability habitats remained aggregated in the hilly terrain of Wendeng and its vicinity, forming multiple high-probability cores in eastern areas such as Huancui and Rongcheng. This spatial configuration suggests that the eastern mountainous-hilly belt constitutes a relatively persistent high-risk zone across the peninsula. By contrast, the northern part of Penglai, coastal lowlands, and flat peri-urban areas exhibited generally low suitability, with a more fragmented and dispersed spatial pattern.

In Qingdao, high-suitability areas were primarily confined to Laoshan Mountain and adjacent hilly terrain. Medium-high suitability also occurred in northern Pingdu, southern Huangdao, and eastern Jimo. Other relatively flat areas were dominated by low-suitability habitats.

### Human exposure risk assessment

Human exposure risk was evaluated by extracting the maximum *H*. *longicornis*-suitability probability within a 500-m buffer of each community (or administrative village). Communities with a maximum probability exceeding the high-suitability threshold (0.78) were classified as high-risk residential areas. Overall, 61.0% (9,893/16,213) of communities in the Jiaodong Peninsula were high risk, with clear regional variation: Weihai (68.8%) > Yantai (67.2%) > Qingdao (51.6%).

Rural areas exhibited the highest proportion of high-risk communities (70.0%), especially in mountainous and hilly townships where high-risk villages were nearly ubiquitous. Urban areas showed the lowest risk (16.9%), while the urban-rural fringe occupied an intermediate position (40.2%). Notably, in “mountain-embedded” cities such as Yantai and Weihai, distinct high-risk zones persisted within urban communities. For example, urban sectors of Qixia (90.9%), Muping (61.5%), and Huancui (45.4%) retained substantial high-risk proportions (Figure 5).

**Figure 5:**
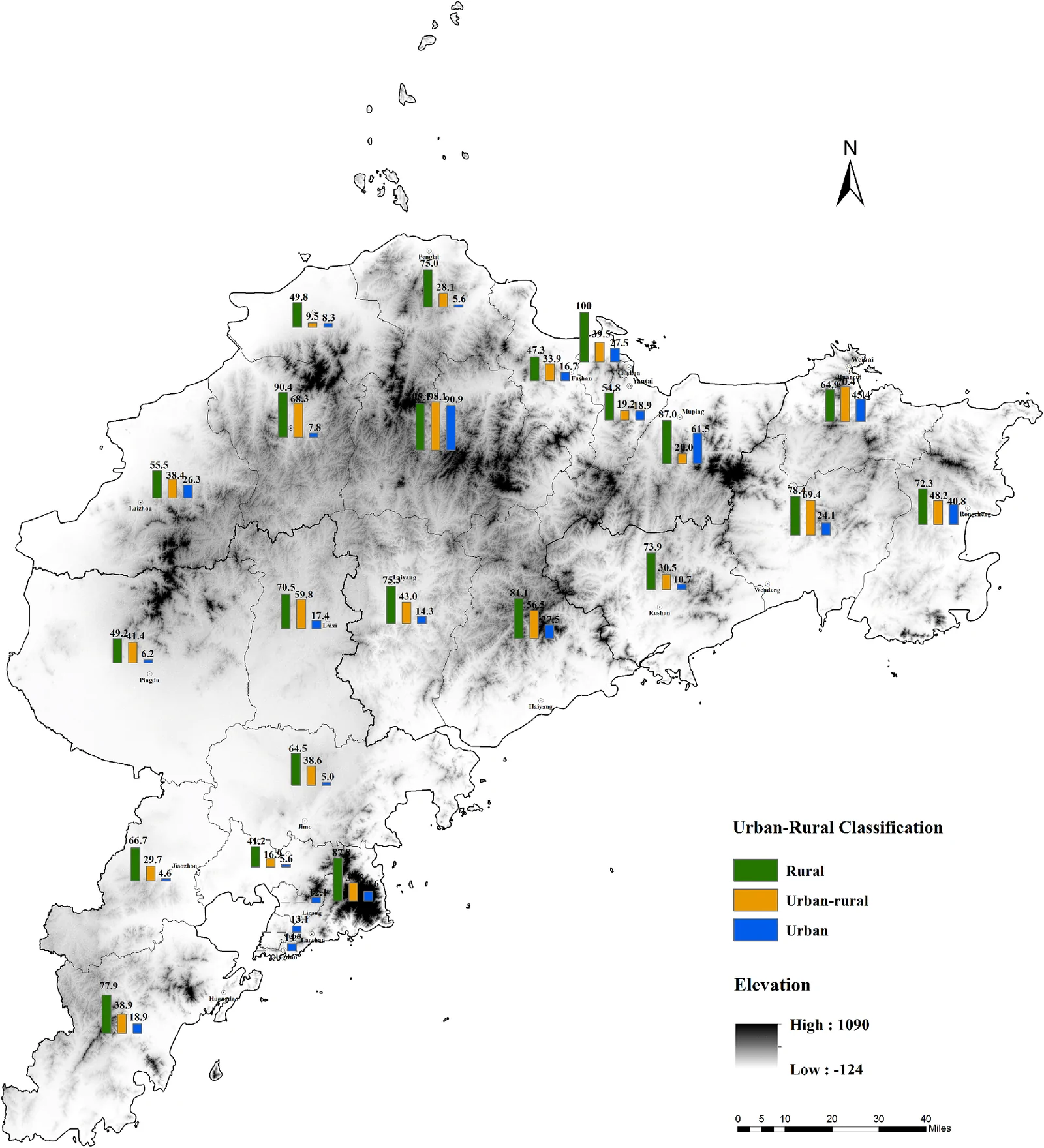
Proportion of high-risk communities classified by urban-rural category across counties and districts of the Jiaodong Peninsula

### Sensitivity analysis

To examine the robustness of *H*. *longicornis*-distribution predictions under different variable-selection strategies, three alternative approaches were implemented and compared with the primary analysis (LASSO combined with Spearman correlation). Both model performance and spatial similarity of output maps were assessed.

In terms of predictive performance, ensemble models constructed under each alternative strategy showed strong discrimination on both the training set (AUC range: 0.8629–0.8825) and the test set (AUC range: 0.8256–0.8453). On the validation set, the VIF-based screening strategy (AUC = 0.8298), the PCA-based forward selection (AUC = 0.8331) and Boruta-Spearman strategy (AUC = 0.8318) yielded robust performance comparable to the primary analysis (AUC = 0.8355) (Table S1).

Spatially, predicted suitability maps from all alternative strategies exhibited high niche overlap and pattern similarity with the primary map (Table S2). Niche overlap (Schoener’s D) ranged from 0.9751 to 0.9826, niche similarity (Warren’s I) from 0.8421 to 0.8682, and pixel-level Pearson correlation of predicted probabilities from 0.9691 to 0.9859. These results indicate that different variable-selection methods did not induce major shifts in predicted spatial patterns, confirming the robustness of the core findings.

## Discussion

Based on field surveys and multi-source high-resolution environmental data, this study employed an ensemble machine-learning framework to generate a high-spatial-resolution prediction of *H*. *longicornis* habitat suitability across the Jiaodong Peninsula. The results demonstrate that LULC type and local vegetation characteristics significantly influence *H*. *longicornis* distribution. The resulting map clearly reveals a spatial pattern characterized by “aggregation along mountain corridors and attenuation toward surrounding lowlands,” with human exposure risk exhibiting pronounced spatial heterogeneity.

### Advantages over previous tick distribution studies

Within China, a few researchers have also used field-survey data to predict tick distributions.^12^^,^^13^ However, these studies often involved a limited number of sampling sites concentrated in a few localities, which may compromise spatial representativeness. By contrast, our sampling strategy—covering more than 2,000 transects distributed across the Jiaodong Peninsula—ensures that survey sites are spatially dispersed, thereby enhancing representativeness and reliability of the resulting suitability map.

Beyond comparisons with previous field-based studies, our approach also offers substantial advantages over the more common practice of relying on historical presence-only records. Such records are often spatially coarse, lacking precise geographic coordinates and frequently approximated to administrative-unit centroids.^8^^,^^9^ This spatial uncertainty precludes integration of high-resolution environmental variables (e.g., land use, vegetation structure) and forces most models to rely on macro-climatic predictors, thereby generating coarse-scale suitability maps with limited local insight. Furthermore, areas with missing or sparse historical data do not necessarily indicate low tick presence probability—they may simply reflect low survey effort. By using field-survey data with exact geographic coordinates and consistent sampling effort across the study area, our study avoids both the spatial aggregation bias and the survey-effort bias inherent in historical records.

### Model performance and robustness

All machine-learning models constructed in this study showed strong discriminative ability on the training set, with RF achieving training AUC values above 0.95. However, the model displayed marked performance decline on the test and independent validation sets (ΔAUC >0.1), indicating clear overfitting and limited generalizability. In contrast, the ensemble model (Ensemble_b)—integrating the gradient boosting machine (GBM), generalized linear model (GLM), multivariate adaptive regression splines (MARS), XGBoost, NNet, and MaxEnt algorithms—delivered the best overall performance on the independent validation set (AUC = 0.8355, Brier score = 0.1533), with only a minor AUC drop between training and validation sets (ΔAUC = 0.0469) and no evident overfitting. This outcome underscores the advantage of ensemble learning in species distribution modeling: by combining predictions from multiple base models, it effectively balances bias and variance, reduces over-reliance on specific data patterns, and yields more robust predictions.^14^^,^^15^

The validation AUC of our ensemble model (0.8355) is comparable to that reported by Gabriel et al. (0.8540),^16^ and outperforms several studies that relied solely on climatic variables or single algorithms.^17^ This highlights the value of integrating high-resolution, multi-source environmental data to characterize local micro-habitat heterogeneity, thereby enabling models to more precisely capture key habitat filters and stressors affecting tick distribution.

The robustness of our approach is further supported by sensitivity analysis. Habitat suitability maps generated under different variable-selection strategies showed high spatial consistency with the primary results (Schoener’s D > 0.97, Warren’s I > 0.84). This indicates that the core findings regarding key environmental drivers and the spatial distribution of high-risk *H*. *longicornis* areas in the Jiaodong Peninsula are not sensitive to the choice of variable-selection method and are thus highly robust.

### Ecological and epidemiological interpretation of environmental drivers

This study reveals that *H*. *longicornis* distribution in the Jiaodong Peninsula is jointly shaped by multi-level environmental factors, including habitat substrate filtering, vegetation-mediated micro-habitat conditioning, anthropogenic and climatic stressors, and topographic-structural features.

First, LULC type emerged as one of the most influential variable, reflecting its role in macro-habitat filtering. Tick occurrence probability was significantly higher in broad-leaved and evergreen forests, which is closely linked to the stable, closed vegetation structures these habitats provide.^18^ Forest environments not only maintain suitable microclimatic conditions (temperature and humidity) but also typically support richer communities of small- and medium-sized host animals, thereby furnishing both the resources and conditions necessary for *H*. *longicornis* survival.^19^^,^^20^

Second, the substantial contributions of summer (ndvi_summer) and the winter NDVI (ndvi_winter) jointly underscore the direct influence of vegetation characteristics on micro-habitat quality. In temperate regions, higher summer NDVI often indicates evergreen vegetation or dense broad-leaved forests with thick litter layers and high canopy closure.^21^ Such vegetation structure offers ticks a more stable, shaded micro-environment, mitigates extreme fluctuations in temperature and humidity, and helps sustain host activity—together creating favorable conditions for *H*. *longicornis* survival.^22^^,^^23^ Similarly, higher winter NDVI reflects the presence of evergreen vegetation or well-structured forests that maintain canopy cover and a stable litter layer even during the dormant season. This persistent vegetation structure alleviates the low-temperature and desiccation risks faced by ticks in winter, while also contributing to a more stable micro-habitat year-round, which may be key to sustaining *H*. *longicornis* populations across seasons.^23^^,^^24^^,^^25^

Third, the negative correlations between *H*. *longicornis* distribution and the nighttime light index (npp) and summer LST (lst_summer) clearly illustrate the dual stressors of human activity and climatic extremes. As a robust proxy of human presence, the negative effect of nighttime light confirms the erosion and exclusion of *H*. *longicornis*-suitable habitats due to urbanization and high-intensity human disturbance.^26^^,^^27^ The adverse impact of high summer temperatures directly reflects the physiological limits imposed by extreme heat on *H*. *longicornis* survival.^28^^,^^29^^,^^30^ In addition, the seasonal standard deviation of (smi_sd) further captures the temporal dynamics of soil water availability. In temperate regions, the wet season provides sufficient moisture to support *H*. *longicornis* development and host activity, while the dry season—if accompanied by a thick litter layer in deciduous forests—can help maintain surface soil moisture and contribute to a more stable micro-habitat year-round, which may be key to sustaining *H*. *longicornis* populations across seasons.^28^^,^^29^^,^^30^

Finally, lower spring VV/VH polarization ratio and lower winter VV/VH polarization ratio generally indicate a dominance of volume scattering associated with complex vegetation structure and high canopy or litter cover, which may enhance micro-habitat suitability for ticks across seasons. In addition, higher spring VH backscatter values generally indicate more complex vegetation structure and greater surface biomass or moisture, which likely provide a favorable microenvironment for *H*. *longicornis* activity in spring.^22^ The positive effects of slope and elevation mainly reflect habitat differences between mountainous and plain areas: mountainous terrain supports more complex vegetation cover, more suitable microclimatic conditions, and relatively low human disturbance, collectively forming a landscape configuration more conducive to *H*. *longicornis* occurrence.^31^^,^^32^

### Spatial pattern and public health implications of predicted *H*. *longicornis* distribution

The prediction map generated by the ensemble model reveals pronounced spatial heterogeneity in *H*. *longicornis* habitat suitability across the Jiaodong Peninsula, exhibiting an overall gradient described as “aggregated along mountain corridors and attenuated toward surrounding lowlands and coastal plains.” This spatial structure aligns coherently with the key environmental drivers identified in this study.

High-suitability areas (predicted probability >0.78) are concentrated in the hilly and mountainous zones of Yantai and Weihai, particularly in Qixia, Muping, Wendeng, and Laoshan, forming a continuous, high-risk corridor of strip-like or patchy configuration. This pattern arises primarily from topographic-driven habitat filtering. Mountainous terrain shapes local climate, soil, and vegetation development, creating the integrated conditions required for *H*. *longicornis* survival: higher elevation and slope are generally associated with denser forest cover (broad-leaved and evergreen forests)—directly corresponding to two of the most influential variables, NDVI and LULC type—which provides a stable, shaded microclimate and diverse host communities.^18^^,^^19^^,^^20^ Moreover, the complex topography of mountainous areas corresponds to lower human activity intensity (reflected in low npp values), reducing habitat disturbance and fragmentation and thereby favoring *H*. *longicornis* population persistence.^26^^,^^33^

Predicted suitability gradually declines from the mountain corridors toward coastal plains, forming medium- and low-suitability zones. This transition is driven mainly by expanding human influence and enhanced environmental stress. In coastal lowlands and areas surrounding towns, higher nighttime light indices reflect strong anthropogenic disturbance and land urbanization, leading to natural habitat loss and fragmentation that directly compress suitable *H*. *longicornis* habitats.^26^^,^^27^ Concurrently, these areas typically experience higher summer land surface temperatures (lst_summer) and simpler vegetation structure, exacerbating heat and drought stress beyond the physiological tolerance of *H*. *longicornis*.^28^^,^^29^^,^^30^

The spatial pattern revealed by our prediction map carries clear implications for public health practice. First, it delineates “key areas” for tick-borne disease prevention in the Jiaodong Peninsula not along traditional administrative boundaries, but along ecological corridors centered on the central mountainous region and radiating into surrounding hills. This suggests that disease-control and forestry authorities should establish cross-jurisdictional surveillance networks across such continuous high-risk corridors and prioritize environmental management interventions—for example, targeted litter removal in high-risk zones. Regarding the public and veterinary health relevance of the tick species in our study area, our prediction map focuses exclusively on *H*. *longicornis*, the dominant species on the Jiaodong Peninsula. *Haemaphysalis longicornis* is a confirmed vector of multiple tick-borne viruses, including SFTS virus (SFTSV) and TBEV, posing direct risks to both humans and livestock. Although *H. flava* has been reported to carry Jingmen tick virus,^11^ both *H. flava* and *H. doenitzi* were collected at low abundances on the peninsula. Therefore, our risk assessment and surveillance recommendations are primarily oriented toward *H. longicornis*, while the other two species still warrant continued monitoring.

Second, integrating the prediction map with human-exposure risk assessment identifies substantial high-risk communities within urban and peri-urban areas of “mountain-embedded” cities such as Qixia and Muping. This challenges the conventional perception of “urban areas as safe zones” and underscores the need for focused risk assessment and public education in residential neighborhoods and recreational green spaces adjacent to natural hills. Finally, the 10-meter resolution spatial information provided by this map can serve as a direct reference for local health units to implement “zoned and tiered” precision control measures—such as deploying tick-monitoring sites within and around high-risk patches, installing warning signs, and conducting targeted behavioral guidance for residents, agricultural workers, and foresters.

### Limitations of the study

Although this study achieved an effective high-resolution prediction of tick distribution in the Jiaodong Peninsula through field surveys and environmental data integration, several limitations warrant consideration in future research.

First, the spatiotemporal coverage and detection reliability of the survey data are constrained. Sampling was conducted during a single summer period, which reflects peak *H*. *longicornis* activity but cannot capture seasonal population dynamics. Moreover, “absence” records obtained via one-time dragging may include false negatives due to the fine-scale spatiotemporal heterogeneity of *H*. *longicornis* occurrence; a single survey can be influenced by weather, sampling timing, and other transient factors, potentially leading to underestimation of suitability in certain habitats and affecting model performance.^34^^,^^35^

Second, the modeling approach remains predominantly environment-driven, with limited incorporation of host-vector interactions. While the study integrated multi-dimensional habitat variables—topography, vegetation, microclimate, and human activity—it did not explicitly include data on the distribution or abundance of key host species (e.g., rodents, birds). Nevertheless, high-suitability tick environments often overlap substantially at the landscape scale with habitats suitable for key hosts (e.g., dense forests provide both shaded microclimates for ticks and refuge/food for small mammals).^19^^,^^21^^,^^27^ Thus, although host data were not directly included, the predicted high-suitability areas likely reflect environments that are concurrently suitable for both ticks and their hosts. Additionally, diurnal and micro-climatic variations during sampling are a potential limitation. Our fieldwork spanned both morning and afternoon, yet we did not record site-specific temperature or humidity. Such fluctuations may affect tick questing and add noise to occurrence data. However, since our study targets broad-scale habitat suitability, not temporal dynamics, major biases are unlikely.

Third, model extrapolation and scale transfer should be approached cautiously. This model was developed specifically for the warm-temperate hilly landscape of the Jiaodong Peninsula, and its predictions are primarily applicable to regions with similar ecological and geographical characteristics. Direct application to areas with markedly different climate, vegetation, or landscape structure would require validation of its transferability. Furthermore, although the 10-meter resolution variables enhanced local prediction accuracy, their use over broader regions may face challenges related to data availability and computational efficiency. Future work could explore a multi-scale modeling framework, employing climate and land-cover data for coarse-grained screening across larger areas, followed by high-resolution refinement in focal zones.

Finally, this study presents the predicted distribution of *H*. *longicornis*. Because only a limited number of occurrence records were available for *H*. *flava* and *H. doenitzi*, we did not conduct separate predictions for these species.

In summary, this study employed an ensemble machine-learning framework based on field surveys and multi-source high-resolution environmental data to delineate the spatial distribution of *H*. *longicornis* habitat suitability in the Jiaodong Peninsula and to identify its key environmental drivers. The ensemble model demonstrated robust predictive performance and effectively captured spatial heterogeneity in *H*. *longicornis* habitat suitability. High-suitability habitats are primarily distributed along mountain and hill corridors, gradually declining toward coastal plains. Vegetation characteristics NDVI are the foremost determinant of *H*. *longicornis* distribution; LULC type follows closely, and the two jointly maintain micro-habitat conditions favorable for *H*. *longicornis* persistence, while human activity and high summer temperatures significantly constrain *H*. *longicornis* occurrence. Human exposure risk exhibits clear spatial heterogeneity, with high-risk communities prevalent in rural areas and even present within certain “mountain-embedded” urban zones.

The high-resolution risk map produced in this study offers a spatially explicit decision-support tool for optimizing tick-borne disease surveillance networks, prioritizing control efforts in key areas, and managing public health risks across the Jiaodong Peninsula. It holds important practical value for advancing the precision prevention and control of regional tick-borne infectious diseases.

## Resource availability

### Lead contact

Requests for additional information and resources should be addressed to the lead contact, Lan-Hua Li (orchid8@sina.com).

### Materials availability

This study did not generate new unique reagents. All tick images used in this study are provided in Document S1, which is submitted along with the manuscript.

### Data and code availability

• All data reported in this study are available within the manuscript and its supplemental information files and are publicly available in the Zenodo repository at https://doi.org/10.5281/zenodo.22322540.
• All code generated for the analysis is publicly available in the same Zenodo repository (https://doi.org/10.5281/zenodo.22322540).
• Any additional information required to reanalyze the data reported in this article is available from the lead contact upon request.

## Acknowledgments

This research was funded by the 10.13039/501100001809National Natural Science Foundation of China (grant no. 81902095), the 10.13039/501100007129Shandong Provincial Natural Science Foundation (grant no. ZR2019MH093), and Prevention and Control of Emerging and Major Infectious Diseases-10.13039/501100018537National Science and Technology Major Project (grant no. 2025ZD01900304). The funders had no role in study design, data collection and analysis, and decision to publish or preparation of the manuscript.

## Author contributions

X.-K.B. participated in field work, extracted and analyzed the data, and drafted the manuscript. S.-P.C., J.-Y.X., H.X., A.-L.X., S.-J.S., H.-R.D., and J.-H.F. participated in field work and data extraction. Y.-Z.C., F.W., and N.T. contributed to data analysis and manuscript revision. Y.L. and L.-H.L. conceived the study, participated in data analysis, and revised the manuscript. All authors read and approved the final manuscript.

## Declaration of interests

The authors declare no competing interests.

## STAR★Methods

### Key resources table

**Table:** 

REAGENT or RESOURCE	SOURCE	IDENTIFIER
**Software and algorithms**		

R Project for Statistical Computing, version 4.4.2	R Foundation for Statistical Computing	https://www.r-project.org/; RRID: SCR_001905
spThin R package, version 0.2.0	CRAN	https://cran.r-project.org/package=spThin
caret R package, version 7.0-1	CRAN	https://cran.r-project.org/package=caret
MLmetrics R package, version 1.1.3	CRAN	https://cran.r-project.org/package=MLmetrics
ranger R package, version 0.17.0	CRAN	https://cran.r-project.org/package=ranger
xgboost R package, version 1.7.8.1	CRAN	https://cran.r-project.org/package=xgboost
earth R package, version 5.3.4	CRAN	https://cran.r-project.org/package=earth
gbm R package, version 2.2.2	CRAN	https://cran.r-project.org/package=gbm
glmnet R package, version 4.1-8	CRAN	https://cran.r-project.org/package=glmnet
nnet R package, version 7.3-20	CRAN	https://cran.r-project.org/package=nnet
dismo R package, version 1.3-16	CRAN	https://cran.r-project.org/package=dismo
terra R package, version 1.8-60	CRAN	https://cran.r-project.org/package=terra
Boruta R package	CRAN	https://cran.r-project.org/package=Boruta
ArcGIS Desktop, version 10.7	Esri	https://www.esri.com/en-us/arcgis/products/arcgis-desktop/overview; RRID: SCR_011081
Google Earth Engine	Google	https://earthengine.google.com/; RRID: SCR_023224

**Deposited data**		

WorldClim version 2.1 bioclimatic variables	WorldClim	https://www.worldclim.org/data/worldclim21.html
Dataset for habitat-suitability prediction of *Haemaphysalis longicornis* in the Jiaodong Peninsula	This paper	Zenodo: https://doi.org/10.5281/zenodo.22322540
Code for habitat-suitability prediction of *Haemaphysalis longicornis* in the Jiaodong Peninsula	This paper	Zenodo: https://doi.org/10.5281/zenodo.22322540
Morphological photographs of tick specimens	This paper	Document S1

### Experimental model and study participant details

#### Sample description

Tick samples were collected by dragging cloth over vegetation at random points across the Jiaodong Peninsula from July to August 2024. No human participants or identifiable personal data were involved in this study.

#### Ethics statement

Ethical approval was not required for this study because it did not involve human participants, identifiable personal data, or experimental procedures involving vertebrate animals.

### Method details

#### Sampling points and sampling method

The random point generation was implemented with the sf and dplyr packages within the R 4.4.2 (key resources table). According to the area of each county-level administrative unit (county, county level city, or district) in the Jiaodong Peninsula, a corresponding number of random geographic points were generated within each administrative unit based on the principle of selecting one random point per 5 km × 5 km grid. From July to August 2024, 2 or 3 transects with tree- or herb-covered vegetation (excluding cropland) were selected near each random point as sampling sites.

Ticks were collected using the standard dragging method: a 1 m × 1 m white cotton cloth was dragged along each transect. Collection was paused every 50 m, and attached ticks were removed into centrifuge tubes. A total dragging distance of 400 m was conducted at each sampling site. The number of collected ticks was recorded, and the geographic coordinates of the transect midpoint were obtained using a GPS device.

Field surveys were conducted by six graduate students, two undergraduate students, and one faculty member with prior field experience in tick surveys (Dr. L.-H. L.). Before the surveys began, Dr. L.-H. L. provided uniform training and field practice to all graduate and undergraduate students.

#### Morphological identification of ticks

All collected ticks were first identified morphologically. To validate the morphological identifications, we selected 10 nymphs and 4 adults of *H*. *longicornis*, 10 nymphs and 4 adults of *H*. *flava*, and 2 nymphs and 2 adults of *H*. *doenitzi* for amplification of the COI gene fragment. The morphological identifications of these 32 ticks were consistent with the COI sequence comparisons. The images of the three tick species are provided Figures S1–S3. Additionally, 37 nymphs with incomplete morphological features were identified directly by molecular methods.^36^

#### Data of environmental predictors

A total of 78 environmental variables were systematically compiled as candidate predictors, covering six categories: climate, topography, land use/human activity, remote-sensing ecological indices, radar backscatter characteristics, and image texture features (Table 2). All variables were resampled to a uniform 10-m spatial resolution by ArcGIS 10.7.

Climatic variables were obtained from the WorldClim database, including 19 bioclimatic variables (BIO1–BIO19) at a spatial resolution of approximately 1-km. During the prediction mapping stage, these variables were resampled to a 10-m resolution to match the spatial resolution of the other environmental layers.

Topographic variables were derived from the 30-m resolution Shuttle Radar Topography Mission (SRTM) digital elevation model (DEM), including elevation, slope, aspect, topographic wetness index, and topographic relief within a 1-km radius. Slope and aspect were obtained in ArcGIS 10.7, while topographic wetness index and topographic relief were obtained in R 4.4.2. Specifically, based on the 30-m resolution DEM, topographic relief was calculated using a 67 × 67 pixel (approximately 1000 m radius) square moving window as the difference between the maximum and minimum elevation within the window. Euclidean distances from each sampling point to the nearest coastline and to the nearest mountain peak were calculated using R 4.4.2 with data from the China National Geomatics Information Platform (CNGIS) and national peak vector datasets, respectively.

Land Use and Land Cover Type data for 2024 were obtained through supervised classification of Sentinel-2 imagery on the Google Earth Engine (GEE) platform.^37^^,^^38^ Nine land-cover classes were distinguished: built-up area, irrigated farmland, greenhouse farmland, roads, water, grassland, broad-leaved forest, evergreen forest, and orchard. Training and validation samples were sourced from field surveys. Seasonal composite images (winter, spring, summer, autumn) were generated from Sentinel-2 scenes with <15% cloud cover. Multiple spectral indices (NDVI, NDWI, NDBI, EVI, SAVI, MSAVI, GNDVI, NBR) were calculated per season, and multi-temporal change metrics (e.g., inter-seasonal NDVI difference, NDVI time-series standard deviation) were constructed to improve discrimination among vegetation types. Classification was performed using a boosted random-forest classifier (100 trees, bag fraction = 0.5).^37^ yielding an overall accuracy of 0.8849 (Kappa = 0.8669). A final 10-m land-use map was produced (Figure S4).

Based on the land-use map, proportional cover of woodland within 100-m and 1000-m buffers centered on each sampling point was computed in R 4.4.2 to characterize local micro-habitat and landscape-level patterns, respectively.^26^ The Nighttime Light Index was used as a proxy for human-activity intensity.

Additional habitat-structure and microclimate variables were extracted from remote-sensing data spanning September 2023 to August 2024. The Normalized Difference Vegetation Index (NDVI) were derived from Sentinel-2 imagery; land surface temperature (LST) was retrieved from Landsat 8 thermal infrared bands. Radar backscatter coefficients (VV, VH) along with the polarization ratio were obtained from Sentinel-1 data. A soil moisture index (SMI) was calculated from Sentinel-1 VH polarization using the normalized difference formula: SMI = (VH - VH_min)/(VH_max - VH_min).^39^ Texture features (complexity and strength) were extracted from Sentinel-2 optical imagery. For each of these variables, we first computed its mean value for each of the four seasons (spring, summer, autumn, winter). Based on the four seasonal means, we then derived the mean and standard deviation to capture seasonal variability and spatiotemporal dynamics.

#### Data preprocessing

All data analysis of this study was completed in R 4.4.2.

To reduce spatial autocorrelation, the R package spThin was used to thin both presence and absence points (*H. longicornis*), retaining only samples >500 m apart (key resources table). All points from Rushan City and Jiaozhou City were set aside as an independent validation set. The remaining points were randomly split into 80% training and 20% testing sets.

This selection was based on ecological and geographical reasoning^40^: Rushan City (eastern hilly terrain) and Jiaozhou City (western plain area) are spatially non-adjacent, represent two contrasting landform types, and belong to different internally homogeneous ecological landscape zones. Using them together as an independent validation set allows testing of model transferability across distinct topographic and ecological gradients.

#### Variable selection

A two-step variable-selection procedure was applied. First, LASSO regression is used for preliminary variable screening, with importance criteria based on the absolute value of coefficients, bias contribution, and direction of impact. The optimal regularization parameter is determined through cross-validation, and non-zero coefficient variables are retained.^41^ Second, Spearman correlation coefficients were calculated among all variables; for pairs with |r| ≥ 0.7, the variable with higher LASSO importance was retained.^42^ The final predictor subset consisted of variables that were both selected by LASSO and exhibited low mutual correlation (|r| < 0.7).

#### Model construction and distribution prediction

Seven machine-learning algorithms commonly used in species distribution modeling were employed: generalized linear model (GLM), multivariate adaptive regression splines (MARS), neural network (NNet), random forest (RF), gradient boosting machine (GBM), extreme gradient boosting (XGBoost), and maximum entropy (MaxEnt).^8^^,^^43^^,^^44^ All models were trained, tuned, and evaluated using the caret package in R. Hyperparameters were optimized via 10-fold cross-validation, maximizing the mean area under the receiver-operating-characteristic curve (AUC). Each final model was refitted on the full training set with optimal parameters.^45^

Variable importance was calculated, and partial dependence plots were produced to visualize marginal effects of key predictors. Spatial prediction was performed using the terra package, generating a continuous habitat-suitability map for potential *H. longicornis* habitats (woodland, grassland, orchard; excluding water, built-up areas, and farmland). Suitability was classified into five levels (low, medium-low, medium, medium-high, high) using the natural breaks (Jenks) method.

### Quantification and statistical analysis

#### Statistical analysis

Model discrimination was assessed using AUC on the training, test, and validation sets; calibration was evaluated with the Brier score. The True Skill Statistic (TSS) was maximized to determine the optimal classification threshold,^46^ based on which sensitivity, specificity, TSS, and F1 score were computed. Overfitting was considered present if the training-test AUC difference exceeded 0.1.^47^ Two ensemble models were built: (1) Ensemble_a combined the three best single models (by validation AUC); (2) Ensemble_b included all single models with AUC >0.75 and no overfitting. Ensemble predictions were generated by averaging predicted probabilities with equal weights. The model with the highest validation AUC was selected for final mapping and interpretation.

#### Sensitivity analysis

Three alternative variable-selection strategies were compared in sensitivity analysis: (a) Boruta feature selection combined with Spearman correlation; (b) variance inflation factor (VIF) screening (threshold >1.9608); (c) forward selection based on principal component analysis (PCA); For each strategy, the same modeling workflow was repeated. Predicted suitability maps were compared with the primary (LASSO - Spearman) map using Schoener’s D and Warren’s I niche-overlap indices,^48^^,^^49^ along with the pixel-wise Pearson correlation between their predicted probability values.

#### Human exposure risk assessment

For each community/administrative village, the maximum *H. longicornis*-suitability probability within a 500-m buffer was extracted. Villages with a maximum probability >0.78 were defined as high-risk residential areas. Village coordinates were obtained via Baidu Map reverse geocoding based on 2023 administrative codes. Using urban-rural classification codes from the National Bureau of Statistics, villages were categorized as urban, urban-rural fringe, or rural. The proportion of high-risk villages was calculated for each county-level administrative unit to examine risk patterns across the urban-rural gradient.

We further re-categorized the original three-digit urban-rural classification codes into three classes (urban, urban-rural fringe, and rural) following the rules below. Code 111 (principal urban area) was classified as urban; code 112 (urban-rural transition zone) as urban-rural fringe; codes 121 (town center), 122 (town-rural transition zone), 210 (township center), and 220 (village) were uniformly classified as rural. For code 123 (special area), the classification was determined on a case-by-case basis according to its actual geographic location and surrounding land-use context (e.g., if located within or adjacent to the main built-up area of a city, it was assigned to urban; if in a peri-urban area, to urban-rural fringe; otherwise to rural).
