# Supplementary material for: High-resolution prediction and risk mapping of Haemaphysalis longicornis habitat suitability in the Jiaodong Peninsula, China
Source: iScience. 2026 Sep 22;29(11):117723. doi: 10.1016/j.isci.2026.117723 (PMC13676737; doi:10.1016/j.isci.2026.117723)
Supplement: Supplementary file 1 [file mmc1.pdf]

iScience, Volume *29*

Supplemental information

High-resolution prediction and risk mapping of *Haemaphysalis longicornis* habitat suitability in the Jiaodong Peninsula, China

Xue-Kun Bian, Shi-Pan Chen, Jia-Yi Xu, Han Xue, Ao-Long Xu, Shi-Jun Song, Ying-Zhi Chong, Fei Wang, Na Tian, Hao-Ran Ding, Jing-Han Feng, Yan Lu, and Lan-Hua Li

Supplemental information
Supplementary Figures

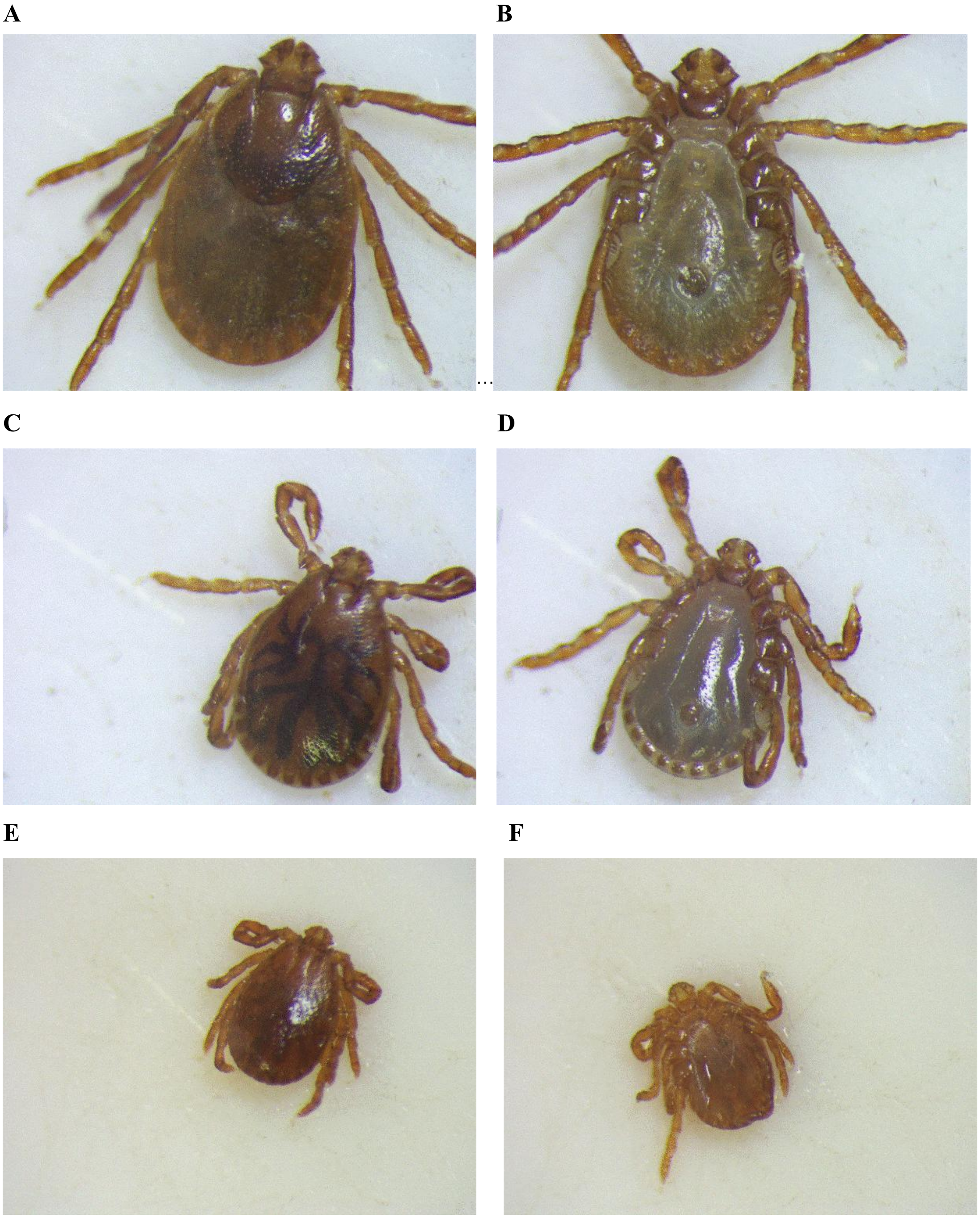

Figure S1. Morphological identification of *Haemaphysalis longicornis* at different developmental stages, related to Figure 1. (A) Back view of the adult female, (B) ventral view of the adult female, (C) back view of the adult male, (D) ventral view of the adult male, (E) back view of the nymph, and (F) ventral view of the nymph.

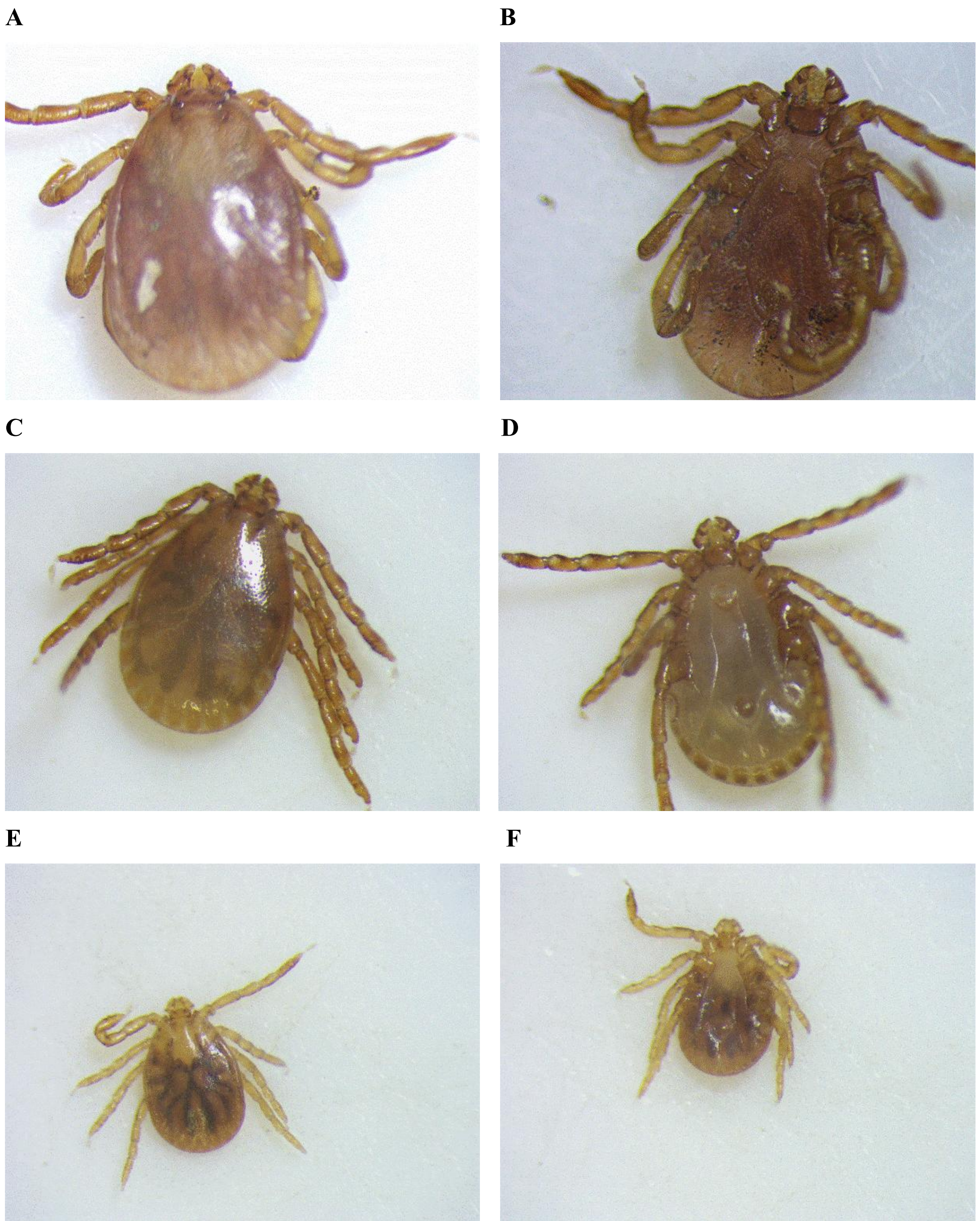

Figure S2. Morphological identification of *Haemaphysalis flava* at different developmental stages, related to Figure 1. (A) Back view of the adult female, (B) ventral view of the adult female, (C) back view of the adult male, (D) ventral view of the adult male, (E) back view of the nymph, and (F) ventral view of the nymph.

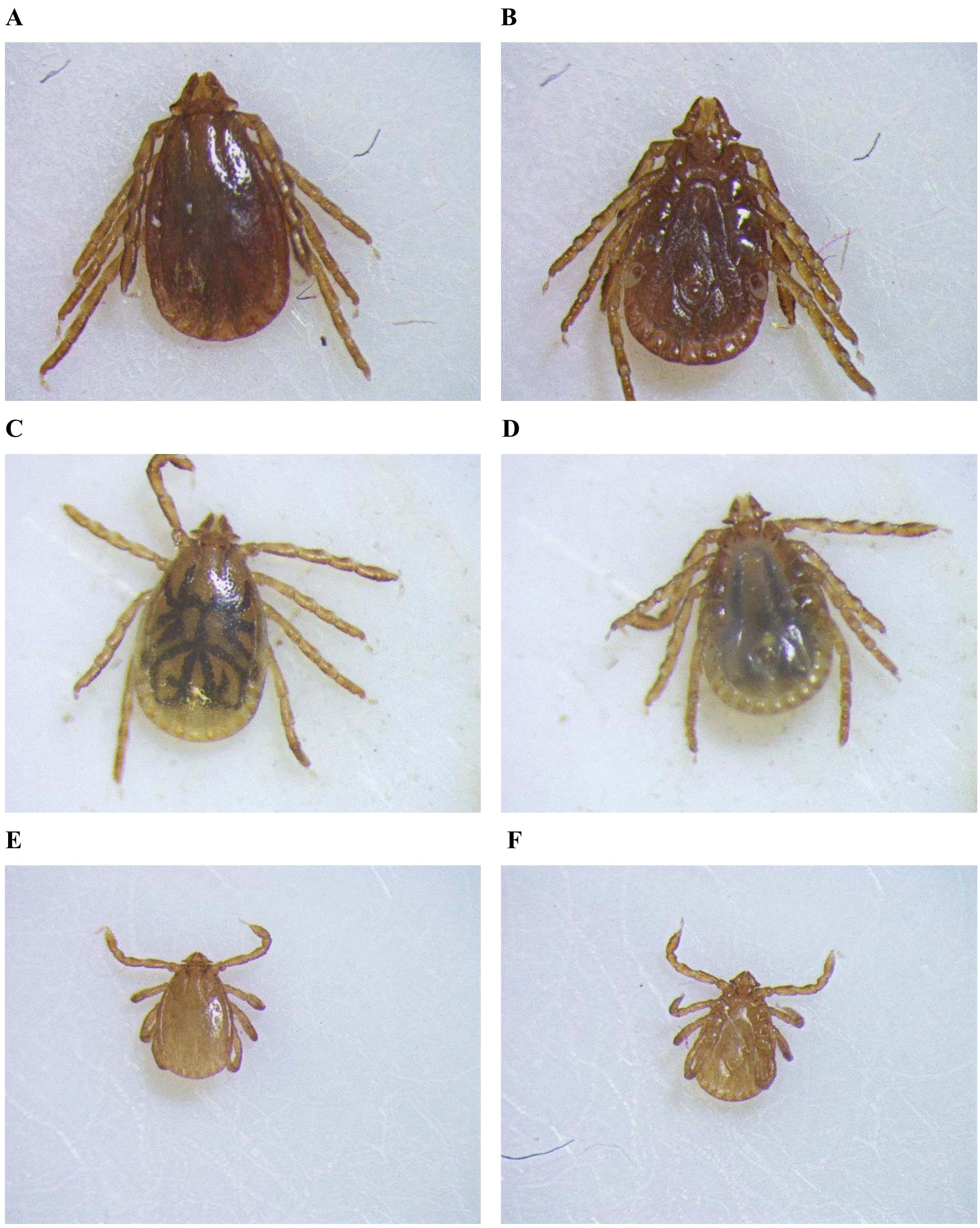

Figure S3. Morphological identification of *Haemaphysalis doenitzi* at different developmental stages, related to Figure 1. (A) Back view of the adult female, (B) ventral view of the adult female, (C) back view of the adult male, (D) ventral view of the adult male, (E) back view of the nymph, and (F) ventral view of the nymph.

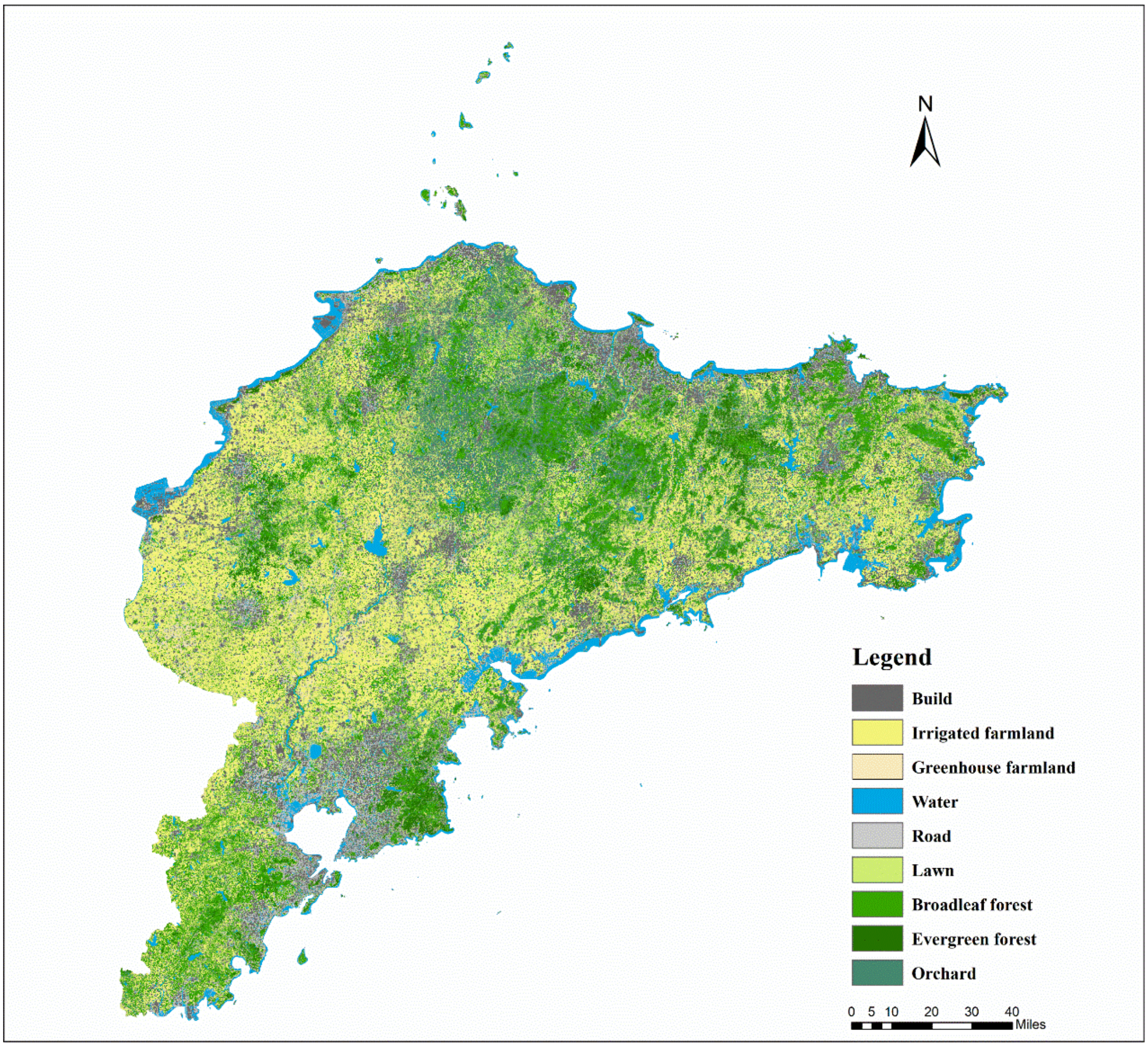

Figure S4. Land use classification map of the Jiaodong Peninsula, related to Figure 4 and Table 2.

Supplementary Tables

Table S1. Model performance of ensemble models under different variable-selection strategies, related to Table 1

**Variable selection strategy**	**Include variables (In order of contribution)**	**Training set**		**Test set**		**Validation set**	
		**AUC**	**Brier score**	**AUC**	**Brier score**	**AUC**	**Brier score**
LASSO Regression Variable Selection (Main Analysis)	ndvi_summer, LULC, ndvi_winter, smi_sd, lst_summer, vvvh_winter, npp, vvvh_spring, vh_spring, slop, Elevation, ts_spring, forest100, TWI, smi_winter, vvvh_autumn, tc_summer, ts_summer, vv_winter, smi_spring, BIO10, lst_winter, tc_spring, BIO18, coastline	0.8872	0.1360	0.8403	0.1593	0.8355	0.1533
VIF for Variable Selection	ndvi_summer, vvvh_spring, vvvh_winter, vh_spring, LULC, lst_summer, ndvi_autumn, ndvi_winter, ts_spring, npp, ndvi_spring, Elevation, vvvh_autumn, slop, tr1000, TWI, lst_autumn, vvvh_summer, forest100, vh_summer, lst_sd, vh_autumn, vh_sd, ts_winter, coastline, tc_summer, tc_sd, BIO11, ts_autumn, ts_sd, tc_winter, BIO10, smi_sd, mount, vvvh_sd, lst_winter, forest1000, tc_autumn, vv_summer, BIO18, lst_spring, aspect, vv_sd, smi_summer, smi_winter, smi_mean, smi_spring, smi_autumn, BIO19	0.8825	0.1389	0.8417	0.1578	0.8298	0.1565

Boruta for Variable Selection	ndvi_summer, LULC, ndvi_winter, lst_summer, npp, vvvh_spring, Elevation, vh_spring, ts_summer, vvvh_winter, ts_spring, forest100, slop, ndvi_spring, TWI, ts_autumn, BIO10, tc_autumn, vvvh_autumn, vv_winter, ndvi_autumn, tc_spring, vvvh_summer, BIO11, ts_winter, lst_sd, lst_mean, tr1000, tc_winter, coastline, mount	0.8764	0.1414	0.8453	0.1565	0.8318	0.1564
PCA for Variable Selection	ndvi_summer, LULC, vvvh_winter, lst_summer, ts_summer, slop, ts_spring, npp, coastline, forest100, TWI, lst_sd	0.8629	0.1481	0.8256	0.1655	0.8331	0.1550

Table S2. Niche overlap and similarity between predicted suitability maps from alternative variable-selection strategies, related to Figure 4

Method	Schoener's D	Warren's I	correlation coefficient (r)
a[a]	0.9826	0.8682	0.9859
b[b]	0.9810	0.8621	0.9838
c[c]	0.9751	0.8421	0.9691

Note: [a] Boruta with Spearman correlation; [b] Variance Inflation Factor (VIF) screening (threshold >1.9608); [c] Forward selections based on Principal Component Analysis (PCA)
